# Dataset on meteorological forcing mechanisms impacting marine circulation and oceanographic variables in the northern part of the Veracruz reef system

**DOI:** 10.1016/j.dib.2023.109637

**Published:** 2023-10-08

**Authors:** José de Jesús Salas Pérez, Adán Guillermo Jordán Garza, David Salas Monreal

**Affiliations:** Universidad Veracruzana, Lomas del Estadio s/n, C.P. 91000, Xalapa-Veracruz, México

**Keywords:** Itinerant low- and high-pressure systems, Surada wind event, Norte wind event, Northern wind circulation, Southern wind circulation, Temperature, Sea level pressure, Biovolume

## Abstract

The dataset is divided in two main groups. The first group, referred to as “Meteorological data”, consists of air temperature, sea level pressure and U and V components of wind direction and intensity: The second group, referred to as “Oceanographic data”, includes biovolume, sea level and water temperature measurements. The meteorological data is derived from model data obtained from the NAAR-NCEP reanalysis for North America, calculated over the area of the Veracruz reef system, Mexico. On the other hand, the oceanographic data was collected in situ using four ADCPs (Acoustic Doppler Current Profilers) anchored at a depth of 20 m at four different reefs within the Veracruz reef system. Both datasets cover a period of 10 days in November 2008, during which successive low-and high-pressure systems occurred over the southwestern Gulf of Mexico. These datasets can be used to evaluate the effect of the pressure changes on marine circulation, residual current and oceanographic variables.

Specifications Table

**Table:** 

Subject	Atmospheric and Marine Sciences
Specific subject area	*Meteorology and oceanography*
Data format	Raw, Analyzed, Filtered Raw and Filtered data: NetCDF (Network Common Data Form) and ascii.txt files
Type of data	Table: geospatial data and time-series
Data collection	***Field data*** Four Acoustic Doppler Current Profilers (ADCPs) were installed in the northern part of the Veracruz reef system within the marine protected area known as “Parque Nacional Sistema Arrecifal Veracruzano” (PNSAV), adjacent to the port of Veracruz [1,2]. These four profilers were deployed on November 10^th^, 2008, and collected data continuously for a duration of 10 consecutive days [1]. This monitoring period encompasses events associated to low-and-high-pressure systems in the western coastal area of the Gulf of Mexico. The four profilers were strategically positioned as follows: two Nortek Aquadop 400 kHz ADCPs on the leeward side of Blanquilla and Anegada Adentro reefs, and two 1 MHz Nortek Aquadop ADCPs on the south side of Isla Verde and on the north side from Isla Sacrificios. The profilers were situated at and approximate depth of 20 m, with a vertical distribution of 2 m and were programmed to record data every 15 min, in addition to a vertical whitening distance of 0.75 m from the surface and 1 m in the bottom [1]. The ADCPs were utilized to measure vertical profiles of the velocity components (u and v) of the marine currents, mean sea level, water temperature and the absolute acoustic intensity. This acoustic intensity was used to estimate plankton biovolumes, following the methodology outlined in [3]. It´s worth noting that only the biovolume of the north-south component of the plankton was considered, as it corresponds to the primary direction influenced by the Surada and Norte wind events. Fluctuations in sea level and water temperature were measured by the ADCPs at the deployment depth [3], coinciding with the maximum depths of the reefs located in the northern area in the PNSAV. From the November 10th to November 29^th^, 2008 [2] itinerants low- and high-pressure systems crossed the study area. The oceanographic parameters were plotted with the original data, which had been filtered with a moving average to eliminate the influence of the diurnal tide in the study area [3]. Additionally, a linear trend analysis was conducted to observe the main pattern of fluctuation during both high- and low-pressure systems.
Data source location	- Institution: Universidad Veracruzana, Facultad de Ciencias Biológicas y Agropecuarias - City/Town/Region: Poza Rica-Tuxpan, Veracruz - Country: México - Latitude and longitude of collected samples/data: Site Point of Oceanographic data:Blanquilla Reef X: - 96.06053 Y: 19.19781Anegada de Adentro X: - 96.06251 Y: 19.22882Isla Verde X: -96.10242 Y: 19.22802Isla Sacrificios X: -96.10628 Y: 19.19859NARR-NCEP atmospheric outputs: X: -150 Y: 0 X1:-70 Y: 80
Data accessibility	Repository name: researchgateData identification number: DOI:10.13140/RG.2.2.23368.29445 Direct URL to data: https://www.researchgate.net/publication/373894440_DIB_JJSP Instructions for accessing these data: • The links above lead directly to the data. After finding the file available, simply click “Download” to access the data, which is compressed in a format .rar

## 1 Value of the Data

• The dataset holds significant value as is encompasses measurements taken during two consecutive wind events, namely the Surada and Norte wind events. The dataset allows for a deeper understanding of circulation reversals within a relatively short time frame of just 10 days, with each event lasting for five days. Consequently, the data reveals the presence of a residual circulation patterns characterized by an anticyclonic submesoscale eddy. Furthermore, the dataset sheds light on the effects of the wind events on marine circulation and their influence on the fluctuations of key oceanographic variables, including water temperature, sea-level and biovolume.
• The dataset can benefit research institutions working on the atmospheric and oceanographic features of the coastal zone of Western Gulf of Mexico.
• For atmospheric studies related to itinerant low- and high-pressure systems and impacts on the climate of Mexico and the Western Gulf of Mexico, the oceanographic dataset proves valuable. This dataset can be used in studies of various aspects, including the analysis of sea surface temperature, comparisons of sea-level records from gauges, and the assessment of biovolume data. Specifically, the biovolume data can be employed to study the composition and dynamics of phytoplankton and zooplankton communities around the reefs of the study area.

## 2 Data Description

The database is contained on a general folder named DIB_JJSP, once you access this folder you will find the structure depicted in Table 1.

**Table 1:** Structure of the folders containing the data on the repository.

Folder 1	Folder 2	Folder 3	Content
Meteorological data	Air temperature		11 .nc files from date 10-11-2008 to 20-11-2008
	Sea level pressure		11 .nc files from date 10-11-2008 to 20-11-2008
	Wind components	U component	11 .nc files from date 10-11-2008 to 20-11-2008
		V component	11 .nc files from date 10-11-2008 to 20-11-2008
Oceanographic data	Biovolume		8 .txt files. Number 1 corresponds to a low-pressure event and number 2 to a high-pressure event. Each text file contains a time series from 10-11-2008 to 20-11-2008.
	Sea level water		8 .txt files. Number 1 corresponds to a low-pressure event and number 2 to a high-pressure event. Each text file contains a time series from 10-11-2008 to 20-11-2008.
	Water temperature		8 .txt files. Number 1 corresponds to a low-pressure event and number 2 to a high-pressure event. Each text file contains a time series from 10-11-2008 to 20-11-2008.

## 3 Experimental Design, Materials and Methods

In-situ data on ADCP acoustic intensity (mesured in dB) were collected over a 10-day period, spanning from November 10 to November 20, 2008, during the fall season. These data were subsequently converted into backscatter intensity and water velocity measurements, covering the depth of 20 m at each anchor site. However, it is important to note that the ADCP data acquired near the water´s surface, within a vertical distance of 0.75 m, were excluded from the analysis due to instrumental noise and contamination from unrelated natural signals that were not relevant to the studied phenomena. Similary, data collected from the water bottom, up to 1 meter within the water column were also not considered. Water velocities and backscatter intensity were recorded using four acoustic Doppler current profilers (ADCPs) strategically moored at a depth of 20 m. The velocity profiles obtained with these ADCPs were divided into cells with a depth resolution of 2 m at 15- min intervals. These instruments were strategically located at the extremities of the reefs within the study area. This placement was based on the hypothesis that the high-frequency circulation patterns around the Veracruz Reef System (VRS) are influenced by wind patterns [4,5], which in turn, should impact plankton biovolumes. Moreover, the currents observed in this region are primarily wind-driven and further rectified by the presence of coral reefs and islands [4]. Data collection was conducted over four 10-day series using two 400 kHz Nortek Aquadop ADCPs, deployed on the leeward side of Blanquilla (BB) and Anegada de Adentro (AA), as well as two 1 MHz Nortek Aquadop ADCPs placed on the south side of Isla Verde (IV) and the north side of Isla de Sacrificios (IS) [1].

## Limitations

‘Not applicable’.

## Ethics Statement

The authors declare that they database completed the ethical requirements for publication in journal Data in Brief. The data do not involve human subjects, animal experiments or the data were collected from social media platforms.

## CRediT authorship contribution statement

**José de Jesús Salas Pérez:** Conceptualization, Methodology, Software, Validation, Formal analysis, Investigation, Data curation, Resources, Writing – original draft, Writing – review & editing, Visualization, Supervision, Project administration. **Adán Guillermo Jordán Garza:** Writing – original draft, Writing – review & editing. **David Salas Monreal:** Writing – review & editing.

## Data Availability

DIB_JJSP (Original data) (www.researchgate.net).

## Appendix Supplementary materials

Image, application 1
